# Mineral Content (Essential and Toxic Elements) of Squid Flesh Is Affected by Maceration with Sodium Salts and Vacuum-Cooking

**DOI:** 10.3390/foods11223688

**Published:** 2022-11-17

**Authors:** Celia Lucas, Faustina Fernández, Sancho Bañón

**Affiliations:** Department of Food Technology and Science and Nutrition, Veterinary Faculty, Regional Campus of International Excellence “Campus Mare Nostrum”, University of Murcia, 30100 Murcia, Spain

**Keywords:** minerals, maceration, cooking, squids, trace metals, cephalopods

## Abstract

Maceration with sodium salts is applied to irmprove water holding capacity in squid-based products. The aim of this work was to determine how the mineral content of squid flesh is affected by maceration and further vacuum-cooking. Atlantic squids (*Loligo vulgaris*) from two fisheries (FAO areas No. 47 and 34) were sampled. Macrominerals (g/100 g) present in raw flesh were Na, Mg, P, S, K and Ca, while microminerals accounting for >1 mg/kg were Zn, Si, Sr, Fe, Cu, Al and Mn. As a result of maceration (3 + 1.5% w: w NaCl+ Na citrate) and vacuum-cooking (at 65 °C for 20 min), some squid minerals was removed. The levels of Cd and As were reduced by half, while Na content increased from 0.28 to 0.49 g/100 g. Maceration with sodium salts generally led to minerals leaching (except for Na) with the medium. Further cooking produced additional losses of most of the minerals present in macerated squid (except Pb and Cd). Squid microminerals were hardly removed with the cooking juice. The consumption of macerated-cooked squid covered > 10% of the recommended dietary intake for Na, P, Zn, Mg and Mn, while health risks were almost negligible and mainly concerned Cd (up to 14% of the probable tolerable weekly intake). The combination of both treatments involves certain loss of most of the essential minerals but also contributes to reducing toxicological risks related to mineral intake through squid products.

## 1. Introduction

The European or common squid (*Loligo vulgaris*) is a cephalopod found in abundance from the Atlantic waters of the North Sea to the west coast of Africa. Currently, this squid is widely exploited by commercial fishing and there is an important flow from Atlantic fisheries in Mauritania and South Africa, among others, to southern European countries, such as Spain, Portugal, Greece and Italy—traditionally the largest consumers of cephalopods [1]. Squid are considered a part of the Mediterranean diet due to their excellent sensory and nutritional properties, including high levels of muscle proteins, ω3 fatty acids and vitamin E [2]. Squid are also a dietary source of essential minerals, such as Ca, Mg, Na, K, P, Zn, Cu, Fe, Mn, Cr and Ni, although they may accumulate toxic heavy metals such as Cd, Hg and Pb [3]. Pollution by As, a metalloid toxic for humans, is perceived as another health hazard linked to squid consumption [4,5]. Mineral accumulation depends on animal traits (species, variety, body size, age, specific tissues and organs) and environmental factors affecting animal diet, in particular, water pollution in fisheries [6]. Squid have weak metabolic capacity [3] because of their short life cycle, and accumulate lower levels of toxic elements than some commonly-consumed fish [7]. Cephalopods captured from offshore sites in coastal sea areas may reach higher levels of heavy metals than others from oceanic fisheries [5,6], being able to accumulate more amounts of heavy metals in the viscera than in the muscle [8] due to their ability to concentrate toxic elements in tissues owing to their carnivorous regimen [9].

Most available data on squid minerals correspond to studies focused on polluting trace metals, without a food technological approach. Mineral content present in raw materials can be affected by treatments applied to obtain squid products (e.g., freezing-thawing, maceration and cooking). Squid caught for international trading are frozen on board as soon as possible as it can take several months for them to be transported and processed in factories. Once there, frozen squid are thawed. Exudates released by thawed squid can be replaced by maceration solutions to obtain a juicer product. Squid can be treated with sodium salts to increase water holding capacity (WHC) before cooking [10]. Sodium chloride is used as a water holding agent, while, in addition, sodium salts of certain organic acids such as citric acid, are used as buffering agents to improve sensory properties [11]. Maceration with sodium salts (in low concentrations) has no preservative purpose as with brined products. Whole squid can be directly macerated or eviscerated to obtain the flesh. Maceration basically provides Na, a debatable nutritional aspect, but can also contain minor quantities of other minerals from powders and tap water. 

The mineral content of macerated raw squid may be affected by cooking treatment. The gain of minerals may result from water loss which occurs during cooking, or possibly from the migration of such elements from the container used, while mineral loss is likely related to their leaching into the cooking juice [12]. Several studies agree that cooking (e.g., grilling, roasting or microwaving) often concentrates the minerals present in seafood and fish products due to dehydration, although there may be exceptions depending on the mineral and the cooking procedure [13,14,15,16,17]. During cooking, protein denaturation promotes structural changes in muscle proteins which reduce WHC [18]. Heating also induces changes in muscle metalloproteins, decreasing their water solubility [19], which may affect leaching of bounded metals. In recent years, vacuum-cooking in plastic bags “*sous vide*” has been increasingly introduced into the industry for manufacturing seafood products. With this method, mild temperatures are applied during more or less time depending on piece size. *Sous vide* cooking at 55–65 °C has been found to be suitable in obtaining squid products of good sensory traits [20], or to prevent microbiological risks in fish fillets kept in refrigeration [21]. Extended cooking contributes to reducing these risks but also increases juice loss, which may negatively affect squid juiciness [20]. 

Maceration offers a good chance to adjust the amount of essential and undesirable minerals in cooked squid products. However, it is unclear how different minerals may simultaneously spread among the squid flesh and the saline solution upon different concentration gradients. Vacuum-cooking may also result in both minerals leaching into their own juices as minerals concentration by dehydration. Accumulative effects of maceration and vacuum-cooking on squid minerals may be antagonistic and should be elucidated. Technological treatments that lead to the loss or gain of minerals can modify the nutritional value and toxicological risks related to mineral intake through squid products. 

The aim was to study how the mineral content of squid flesh is affected by maceration and further vacuum-cooking. Dietary intake for minerals and toxicological risks for As and heavy metals through the consumption of squid flesh were also determined.

## 2. Materials and Methods

### 2.1. Squid Obtention and Processing

Fishing zones and the time course followed for obtaining squid are indicated in Table 1. Squids were caught using trawl nets in two different fishing zones from the Central-Eastern Atlantic Ocean: (i) FAO No. 34 (Mauritania); and (ii) FAO No. 47 (South Africa) [1]. Fished whole squids were arranged in blocks, packed in polyethylene bags and then frozen on board at −18 °C, placed in cardboard boxes and kept at −18 °C during storage and further transport to a cephalopod products factory (New Concisa S.L, Cieza, Murcia, Spain). 

Manufacturing stages included: frozen storage, thawing, draining, washing, maceration, draining, eviscerating, washing, draining, vacuum-packing, cooking and freezing. Squid blocks were thawed by immersion in a tank containing a water solution with 5 mg H_2_O_2_/kg (at 4 °C for 24 h). Thawed squids were drained and macerated by immersion in a tank containing squid and maceration solution at 1/3 w:w (at 10 °C for 24 h) with product tumbling every 3 h. A commercial maceration solution containing NaCl (3% w:w) + citric acid/sodium citrate (1.5% w:w) was prepared with tap water (Budenheim Iberica, El Puig, Valencia, Spain). The mineral content of the maceration solution was checked using ICP-OES (rest of minerals) and ICP-MS (Cd, Pb and Hg) systems (see methodology in 2.4 subsection) (Table 2). Macerated whole squid were drained again and manually eviscerated to obtain the edible flesh (excluding head and viscera). Squid flesh was washed with water, drained and packed under vacuum (10 mbar) into a TMPly Cook-in CN2000 cooking bag (Cryovac, Sant Boi de Llobregat, Barcelona, Spain) composed of eight layers of polyethylene (70%) and polyamide (30%) using a TFS200-MSV machine (ULMA Packaging, Gipuzkoa, Spain). Vacuum-packed squid (squid units without viscera and non-edible parts of 226 ± 24 g) was cooked at 65 °C for 20 min in an MC-1000 kettle heated with propane (Palinox, Sabadell, Barcelona, Spain). Cooking endpoint, juice loss and microbial load were previously tested in the factory (data not shown). Internal temperature was checked using a probe through the body cavity of the squid’s mantle. The bags with cooked squids were kept at −18 °C for 1.5 h in a TCE-800 spiral tunnel (Tameinsa, Cambre, A Coruña, Spain). The final frozen product was placed in cardboard boxes and kept at −18 °C for up to 2 months before sampling. Average (±SD) variations in product weight (g/100 g) after each manufacturing stage were: (i) thawing: −2.3 ± 0.2; (ii) maceration: +19.6 ± 2.3; (iii) evisceration: −27.9 ± 1.9; and (iv) cooking: −23.3 ± 2.1. For sampling, approximately 1 kg of squid (six eviscerated pieces) from the same block was homogenized in a 1.6 cutting machine (Taurus, Lérida, Spain), placed in plastic jars and frozen at −18 °C until further analyses. Wet samples were analyzed.

### 2.2. Proximate Composition, Volatile Basic Nitrogen and pH

The following were determined: Proximate composition according to the respective International Standard Organization procedures [22]. Moisture content (g/100 g) after dehydration using a D6450 drying oven (Heraeus, Boadilla del Monte, Madrid, Spain) and a BP 110S scale (Sartorius, Alcobendas, Madrid, Spain). Total lipids (g/100 g) by Soxhlet extraction using a 4002841 Det-Grass extraction unit (Selecta, Barcelona Spain) and petroleum ether as solvent. Ash content (g/100 g) by incineration using an HK-11 muffle furnace (Forns Hobersal, Caldes de Montbui, Barcelona, Spain). Total nitrogen (TN) (g/100 g) with the Kjeldhal method. A K-435 digestion unit (Büchi Labortechnik, Flawil, Switzerland) and a KT 200 Kjeltec distillation unit (Foss, Barcelona, Spain) were used. Ammonia was titrated with hydrochloric acid 0.1N using an automatic Titrino 702 SM instrument equipped with a No. 6.0233.100 combined electrode (Methrom Schweiz, Zofingen, Switzerland). Factor 6.25 was used to convert nitrogen into protein. Total volatile basic nitrogen (TVBN) according to a specific procedure using hydrochloric acid 0.01N [23]. The pH by using a HI-98129 pH meter (Hanna Instruments Gipuzkoa, Spain) equipped with a combined electrode, Cat. No. 52–22 (Ingold Electrodes, Wilmington, DE, USA).

### 2.3. Determination of Minerals

Samples were prepared according to the EPA Method 3015, “A microwave assisted acid digestion of aqueous samples and extracts”. Then, a 0.20 g sample was added to a 25 mL digestion tube together with 4 mL of concentrated nitric acid (68 g/100g purity) and 1 mL hydrogen peroxide water solution (33% purity) for subsequent digestion in a microwave oven. The following were also added to the Teflon reactor: 300 mL ultrapure water, 30 mL hydrogen peroxide water solution (33 g/100 g purity) and 2 mL concentrated sulphuric acid (98 g/100 g purity). The temperature ramps and pressure used during sample digestion were initially, 20 °C and 40 bar, increasing at 10 bar/min for 30 min up to 220 °C and then maintained at 220 °C for 20 min. After completing digestion, the Teflon reactor was cooled and decompressed to ambient temperature and pressure to obtain the mineralized sample, which was diluted with ultrapure water using double gauge tubes of 10 mL (microminerals) or of 25 mL (macrominerals). Squid minerals (except Hg) were analyzed by Inductively Coupled Plasma Optical Emission Spectrometry (ICP-OES) [24] in a Thermo ICAP 6500 Duo apparatus (Thermo Fisher Scientific, Waltham, MA, USA). Calibration standards were prepared with a solution containing thirty-one minerals (SCP Science, Quebec, Canada) and ultrapure water. A Standard Reference Material 1577c (Bovine Liver; National Institute of Standards and Technology, USA) was used for method validation. For more details, see Fernández et al. [25]. Each mineral determination was performed at specific wavelengths ranging from 167.1 to 670.8 nm. The sample concentrations of minerals were calculated as follows.

(1) $$C=\frac{S\;x\;D}{W}$$

where C was the content of macro- (g/100 g) and microminerals (mg/kg) in the squid sample, S was the mineral concentration of the working solution, D the dilution factor and W the sample weight. The quantification limits (LoQ) were: 0.01 g/100 g (Na, K, Mg and Ca); 1 mg/kg (Al, Fe and Si) and 0.01 mg/kg (rest of minerals). These LoQ were considered suitable for studying mineral diffusion (levels of up to 0.9 g/100 g) and toxic trace metals (authorized levels of up to 1 mg/kg) in squid flesh [26]. All mineral determinations had suitable values of linearity (r^2^ ≥ 0.99), repeatability (± 10%) and recovery percentages ranging from 94.6 to 108.9%.

Pb, Hg and Cd were analyzed in mineralized samples using a 7900 single quadrupole ICP mass spectrometer (ICP-MS) (Agilent Technologies, Las Rozas, Madrid. Spain) system with a Peltier-cooled Scott-type nebulizer chamber, a MicroMist concentric nebulizer, nickel cones, a 27.12 MHz radio frequency generator and a 1600 W Fassel-type quartz torch, argon mass flow control in plasma, auxiliary line, adjustment line and carrier gas, a hyperbolic quadrupole mass filter (3 MHz and 2–260 amu) and a simultaneous digital/analog detector with nine orders of magnitude of linear dynamic range and a collision/reaction cell. Mineral isotopes selected were: 111Cd, 202Hg and 208Pb. The LoQ for the three elements were lower than 0.01 mg/kg. Gallium and rhodium were used as internal standards. For more details concerning the analytical procedure (calibration, reproducibility and reliability of results), see Motas et al. (2021) [27].

### 2.4. Calculation of Dietary Indexes for Minerals

Dietary Reference Intake (DRI) was calculated according to the European Food Safety Agency (EFSA) guidelines [27,28,29] for an adult person (male and female older than 19 years) with no special nutritional requirements. References used were: (i) Population Reference Intake (PRI): level of (nutrient) intake adequate for virtually all members of a population group. This meets the requirements of 97.5% of individuals in the population; (ii) Adequate Intake (AI): the average observed or experimentally determined approximations or estimates of nutrient intake by a population group (or groups) of apparently healthy people assumed to be adequate. It is the value estimated when a PRI cannot be established; (iii) Tolerable Upper Intake Level (UL): the maximum level of total chronic daily intake of a nutrient (from all sources) judged unlikely to pose a risk of adverse health effects to humans.

The Estimated Weekly Intake (EWI) for As, Pb, Cd and Hg through the consumption of cooked squid was calculated as follows:

(2) $$\mathrm{EWI}=\frac{C\;\times \;\mathrm{FIR}\;\times \;7}{\mathrm{WAB}},$$

where EWI is the Estimated Weekly Intake in μg/kg b.w., C is the metal concentration in seafood (μg/g w.w.); FIR is the Food Ingestion Rate for cephalopods (9.8 g per person and day); 7; gives the expression of results of a weekly basis; and WAB is the average consumer body weight (60 kg) [14].

The Probable Tolerable Weekly Intake (PTWI) for toxic elements is established in 15 (As), 2.5 (Cd), 7 (Hg) and 25 (Pb) μg/kg b.w [30,31,32,33].

### 2.5. Experimental Design

A randomized factorial design with two treatments (maceration and cooking) was performed for the study. Sample size was n = 72:3 squid blocks × 2 (FAO 34 and 47) × 2 (untreated and macerated) × 2 (raw and cooked) × 3 manufacturing batches. The effects of both treatments on the dependent variables were specifically determined in the FAO 47, FAO 34 and whole (FAO 47 + 34) sample using a two-way ANOVA (Tukey range test; *p* < 0.05 significance level). Data were analyzed with the Statistics 8.0 for Windows software (Analytical Software, Tallahassee, FL, USA).

## 3. Results

### 3.1. Differences in Squid Composition and pH

Data on squid composition and pH are shown in Table 3. There was an interaction between maceration and cooking for all compositional traits. Maceration involved different changes in squid (FAO 47 + 34) composition: (i) moisture content increased in both raw (from 79.83 to 84.00 g/100 g) and cooked squid (from 76.83 to 79.54 g/100 g) to the detriment of other components; (ii) protein content decreased in both raw (from 17.34 to 12.63 g/100 g) and cooked (from 19.28 to 15.16 g/100 g) product; (iii) TBVN level increased in the raw (from 10.61 to 14.72 mg/100 g) but not (around 17 mg/100 g) in the cooked product; (iv) lipid content was similar in the raw (around 1.9 g/100 g) while increased in the cooked product (from 3.01 to 3.59 g/100 g); and (v) ash content increased in both raw (from 1.53 to 1.76 g/100 g) and cooked squid (from 1.66 to 2.59 g/100 g). As expected, maceration improved WHC, increasing ash content at the same time. Maceration slightly increased the pH in raw squid, although, once cooked, the pH remained around 7.1 for all treatments. In general, the above-mentioned effects reproduced when the FAO 47 and 34 samples were separately analyzed, despite FAO 47 squid having a higher wet base.

### 3.2. Changes in Mineral Content during Squid Processing

**Figure 1:**
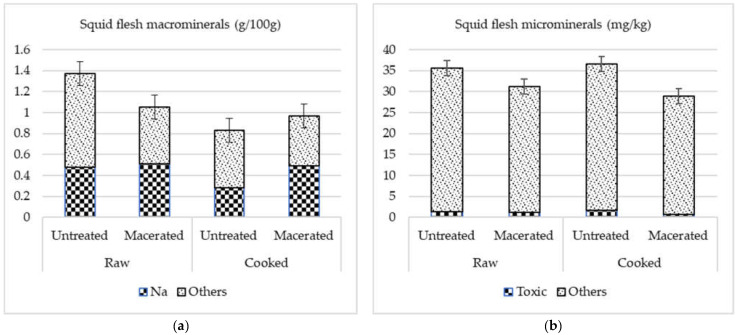
Total content of macrominerals (**a**) and microminerals (**b**) of raw, macerated and cooked squid flesh from two Atlantic fisheries.

Mineral contents of raw and cooked squid flesh are shown in Table 4. Overall (FAO 47 + 34), the most abundant macrominerals in raw and cooked squid were Na, followed by far by P, S and K. There was interaction between maceration and cooking effects for all macrominerals, except Ca. In raw squid, maceration resulted in a product with more Na (from 0.33 to 0.80 g/100 g), less K (from 0.23 to 0.08 g/100 g), P (from 0.28 to 0.15 g/100 g) and S (from 0.30 to 0.22 g/100 g), and with similar levels of Mg (around 0.04 g/100 g) and Ca (around 0.02 g/100 g). In cooked squid, maceration resulted in a product with more Na (from 0.33 to 0.45 g/100 g), less K (from 0.17 to 0.08 g/100 g) and similar levels of Ca (around 0.02 g/100 g), Mg (around 0.03 g/100 g), P (around 0.15 g/100 g and S (around 0.19 g/100 g). As shown in Figure 1, Na content was higher in the raw than in the cooked squid, so that a part of the Na gained by squid flesh during maceration passed to the cooking juice.

Overall (FAO 47 + 34), the most abundant (>1 mg/kg) microminerals determined in raw and cooked squid were, in decreasing order: Zn, Si, Sr, Fe, Cu, Al and Mn, while squid levels of toxic elements were lower, with maximum values of 1.28 (As), 0.32 (Cd), 0.07 (Pb) and 0.01 mg/kg (Hg). There was interaction between maceration and cooking effects for As, B, Cd, Hg and Mn. In raw squid, maceration decreased levels of As, Cd, Cu, Mn, Rb and Zn, while not affecting levels of the rest of the microminerals. In cooked squid, maceration decreased levels of As, B, Cu, Mn and Rb, and did not affect the rest of the levels. A relevant aspect was that levels of Cd and As were decreased when cooked squid was previously macerated (from 0.32 to 0.17 mg/kg for Cd and from 1.28 to 0.97 mg/kg for As). Results obtained in the respective FAO 47 and 34 samples were generally quite similar. The total content of microminerals (including all those with concentrations ranging 0.01–1 mg/kg) was quite similar in the raw and cooked squid.

### 3.3. Dietary Intake and Consumer Risk

Dietary intake through consumption of cooked squid was calculated for selected minerals (Table 5). Overall (FAO 47 + 34), the consumption (100 g) of cooked product by an adult consumer (60 kg) covered a relevant part of the DRI established for Na (17–28%), P (20–21%), Mg (9–12%), Zn (10–12%) and Mn (8–9%), while the percentage of DRI covered for other minerals such as Ca, K, As, Cu or Fe was much lower. Maceration hardly modified squid mineral intake except for Na and Mg, the DRI of which increased by up to 11% and decreased by up to 4%, respectively.

Consumer risk of toxic elements linked to cooked flesh squid intake (Table 6) was acceptable and mainly concerned As and Cd. Overall (FAO 47 + 34), maceration reduced the EWI for As and Cd from 1.38 (9.8% PTWI) to 0.50 µg/kg b.w. (3.5% PTWI), and from 0.36 (14.4% PTWI) to 0.20 (7.8% PTWI) µg/kg b.w., respectively. These EWI for As and Cd were slightly higher in the FAO 47 than in the FAO 34 product. In contrast, the EWI calculated for Hg (0.01 µg/kg b.w.) and Pb (0.06–0.09 µg/kg b.w.) were irrelevant (<0.4% PTWI) for this Atlantic squid flesh. 

## 4. Discussion

Fishery origin led to some differences in Na, K, Mg and other squid minerals. As mentioned, the quantities of minerals accumulated in cephalopods may vary depending on animal (species, variety, body size, age, specific tissues and organs) and environmental factors (animal diet and water pollution in fishery) [6]. Among these, animal diet would be particularly important, since squid is a carnivorous species with a low metabolic capacity and a certain tendency to accumulate minerals [3,9]. Therefore, it is expected that the composition of raw materials from distant fisheries may vary despite squid size being standardized. Na was by far the most abundant mineral in squid flesh, as others have reported [3,25], due to sodium chloride being the predominant salt in marine waters. Squid flesh also contained relevant levels of P and S, two elements that integrate several salts, proteins, phospholipids and other molecules. Marine waters and sediments may also contain other salts, so squid can accumulate little quantities of other minerals, such as Ca, Mg, Zn, Cu, Fe or Mn, among others [3]. 

Minerals can be removed from raw material through thawing exudates, maceration effluents and/or cooking juices. The freezing–thawing cycle may cause some denaturation of muscle proteins [35], diminishing WHC which favors the exudation of liquids containing dissolved minerals. In the present study, thawed squid were drained to control microbial loads, and exudates were removed. Maceration with sodium salts improved WHC in squid flesh by way of an osmotic retention mechanism. Sodium chloride acts as the main water holding agent [10,36], while sodium citrate is a buffering agent which is able to move the pH away from the isoelectric point of muscle proteins, which facilitates their rehydration [37]. In fact, the pH slightly increased when squid was macerated, likely due to the buffering action of sodium citrate and the formation of trimethylamine from protein hydrolysis [38]. Maceration mainly increased Na content, favoring water retention. As seen, Na and moisture contents of raw squid flesh were increased by 0.41 g/100 g and 4.2 g/100 g, respectively. This fact evidences the relevance of maceration with sodium salts for the resulting WHC in this product. Guldas and Hecer [11] reported weight increases of 4.0–4.7% when macerated squid flesh with citric acid (3.5% w:w) and NaCl (2% w:w). Na diffusive behavior was a logical exception compared to those seen for other minerals that had lower concentrations in the maceration medium than in squid flesh. Maceration increased squid wet base and favored minerals leaching. The result was a decrease in the levels of most macro and micro squid minerals with respect to the thawed raw material. For example, Zn level decreased from 11.63 mg/kg (untreated) to 8.23 mg/kg (macerated) in raw squid treated with a solution containing 5.50 mg Zn per kg. This was the most common diffusive pattern for squid minerals, although with some exceptions, such as for Al, which was more concentrated in the medium than in squid flesh. In any case, the presence of minor minerals in the maceration sodium salts does not seem to play a relevant role in the mineral content of macerated raw squid. Squid flesh minerals have been studied in Chinese (*Loligo chinesis*), Pacific (*Ommastrephes bartramii and Dosidicus gigas*), Atlantic (*Illex argentines*), Mediterranean (*Loligo vulgaris*) and Japanese squid [5,14,15,39]. Despite existing differences in body weight and origin, from these studies it can be extracted that Zn (7.1–14.8 mg/kg), Fe (6.0–10.0 mg/kg) and Cu (0.8–5.5 mg/kg) are the predominant microminerals in squid flesh, as also determined in the present study. Concentrations (mg/kg) of toxic metals reported by the above authors for raw squid were Cd (0.02–1.28); Hg (0.01–0.05), Pb (0.03–0.10) and As (2.1–22.0). The Atlantic squid analyzed in the present study would be at the bottom of these ranges. Available data are scarce on how squid minerals are affected by maceration with sodium salts. There are basically two maceration methods, immersion in a tank appropriate for small pieces, and injection is used in large pieces, as with giant squid, due to the transference ratio for sodium salts being more efficient. A product vacuum-cooked in its own juice will require more intense maceration than one cooked in a salted broth. In addition, agents that improve juice retention, such as phosphates, carrageenan and others, can be used [11]. In a previous trial [25], a maceration solution containing 13 g sodium salts per kg (3.1 g Na/kg) was injected into giant squid arms that were then cooked in a salty broth. As a result, injected raw arms only reached 0.15 g Na/100 g, a lower Na level than those reached in the present study. Both trials agree that raw flesh from Atlantic (macerated or not) and Pacific (macerated) squid had a similar micromineral profile: a predominance of Zn over other metals and a scarce presence of toxic metals.

Changes in the mineral content of squid flesh due to heating are closely related to minerals leaching with the cooking juice and/or flesh dehydration [12]. Vacuum cooking at mild temperatures for long periods aims to retain as much juice as possible and to reach a suitable gelatinization of collagen to obtain an edible texture. A culinary study where *sous vide* squid was cooked at 45 °C, 53 °C, and 72 °C for 20 min found that intermediate conditions are sufficient to obtain a product with good sensory traits [20]. This may be due to denaturization of myosin and collagen occurring at mild temperatures (50 °C and 57 °C, respectively) in squid muscle [40]. Microbiological risks would also be controlled in refrigerated *sous vide* products. For example, vacuum-cooking at 65 °C for 5 min was sufficient to inhibit pathogen bacteria (e. g. *S. aureus, B. cereus, C. perfringens* and *L. monocytogenes*) in salmon fillets kept at 2 °C for 45 days [21]. Thus, the cooking conditions used in the present study (65 °C for 20 min) should ensure microbial quality in a cooked-frozen squid product. In fact, the microbial quality of cooked squid was checked (data not shown) in the factory. In addition, *Sous vide* cooking conditions moderated juice loss. For example, a giant squid arm may lose around 70% of its raw weight when cooked in polypropylene bags at 100 °C for 10 min [41]. As seen (Figure 1), cooking decreased the concentration of macrominerals but, in contrast, did not affect the concentration of microminerals in the untreated squid. This suggests that squid microminerals leach with some difficulty with cooking juice, perhaps because product–juice diffusion gradients are less effective as they are present in microquantities. 

Maceration and cooking would have associated effects on the mineral profile of squid flesh. During vacuum-cooking, a part of Na passed from squid to the cooking juice, remaining in the bag. This fact would be proportional to the quantity of Na retained by squid. As seen, Na content decreased less in the untreated (0.07 g/100 g) than in the macerated (0.26 g/100) cooked squid. Despite this, maceration fullfed its technological aim: moisture content (juice retention) increased by 2.7 g/100 g in the ready-to-eat product when macerated. On the other hand, it can be deduced that a part of the macro (K, Mg, P and S) and microminerals (As, B, Cr, Mn and others) whose levels decreased with cooking, ended up in the cooking juice. For example, the noticeable loss of P and S reveals that a part of the muscle component was removed with the cooking juice. In contrast, other squid minerals such as Zn or Cd were concentrated with cooking. This different behavior (dilution of concentration) might be due to several causes. Heating coagulates muscle proteins, which favors juice exit and increases dry basis [8]. This may concentrate squid minerals or not, depending on the quantity of minerals lost with the cooking juice. Moreover, cooking decreases the solubility of metalloproteins and may induce different chemical reactions [19], therefore coagulated proteins would have more difficulty in passing to the juice released by squid. Cd levels in squid mantle may considerably increase by roasting and industrial canning [42]; this behavior is associated with changes in metallothioneins during squid processing, while, in contrast, Pb content is not dependent on processing or associated with metallothioneins. Thus, processing operations may affect Cd and Pb content differently due to the specific metal bioaccumulation and the chemical features of each heavy metal type. This would explain why the levels of some metals can increase or not with cooking. Macerated or not, any change in the mineral content of squid flesh will depend upon cooking conditions (time, temperature and cooking procedure) [43]. Different studies reported that mineral content increases with cooking in fish and seafood products [13,14,15,16,17]. There is consensus that Zn, Fe and Cu were the most abundant trace metals in cooked squid flesh. A previous trial on macerated squid arms also revealed that cooking (in a salted broth with 0.56 g Na/100 g) increases levels of Na (from 0.15 to 0.37 g/100 g), Zn (from 13.7 to 21.4 mg/kg), As (from 0.48 to 0.81 mg/kg) and Cd (from 0.02 to 0.09 mg/kg) [25]. Evidently, the different cooking conditions tested (cooking in salted broth, microwaving, grilling or pan-frying) may explain these findings. In the present study, macerated squid was cooked at a mild temperature (65 °C), which moderated juice loss. Concentrated or not, the levels of Cd, Hg and Pb determined for the ready-to-eat product were well below the maximum levels for Cd (1 mg/kg) and Pb (0.3 mg/kg) authorized in cephalopods flesh and for Hg (0.3 mg/kg) authorized in fishery products [26]. As content was also below the maximum limits permitted for fish and shellfish in Australia (1–2 mg/kg) [44].

The consumption of cooked squid covered a relevant percentage (>10%) of the DRI established for Na, P, Zn, Mg and Mn. Other studies agree that cooked squid is a relevant dietary source of Na and Zn [14,19]. As expected, Na intake was higher (up to 28% DRI) when cooked squid was previously macerated. This percentage can even be increased if vacuum-cooked squid is consumed with its own juice. In a previous trial, macerated-cooked giant squid arms provide less dietary Na (16% DRI) [25]. Na intake associated to the consumption of macerated-cooked squid poses a health risk and could negatively affect its consumption. The close relationship between hypertension and dietary sodium intake is widely recognized and supported by several studies [45]. Regarding toxic metals, squid flesh from the FAO 34 and 47 fishing zones can be consumed with a wide margin of safety. The %PTWIs calculated in the present study for Cd, Hg and Pb were below recommendations established by the EFSA [31,32,33]. Similarly, their levels of inorganic As were below the PTWI (15 μg/kg b.w.) and the range (0.3–8 μg/kg b.w.) of benchmark dose lower confidence limit published by the EFSA [30]. Both evisceration and the possibility that offshore marine waters are less polluted may support this finding. However, it has been noted that the health risk linked to the intake of toxic metals also depends on other factors affecting their bioaccessibility (e.g., denaturation and de-methylation of muscle metalloproteins) [46]. Other studies [14,15,38] agree that the consumption of squid flesh does not pose any health risk to consumers; the % PTWI reported in these studies range from 7.2 to 35.2% (Cd), 0.2 to 0.3% (Pb) and 0.1 to 2.9% (Hg). It is also known that cephalopods tend to accumulate more Cd than Hg or Pb [46]. The results obtained support the idea that consuming products based on squid flesh does not have to be associated with the intake of toxic metals and semi-metals. The whole technological treatment (evisceration, maceration, cooking, etc.) applied to the squid leads to a reduction in the intake of mineral pollutants, which might even be modulated by modifying the processing conditions (adjusting sodium salts concentrations and/or replacing sodium salts by other water holding agents in maceration media; using agents to retain cooking juices). Sodium intake through the consumption of squid products could also be reduced by applying some of these strategies.

## 5. Conclusions

Mineral content influences some important technological (WHC) and nutritional (dietary intake and health risks) properties of squid-based products. Raw materials from the two Atlantic fisheries studied have no marked differences as regards mineral profile. Maceration with sodium salts and vacuum-cooking strongly affects the mineral content in squid flesh. Maceration involves squid flesh absorbing more Na but other minerals will be leached from the raw material. As a result of maceration and cooking, some squid minerals are removed, particularly the Na gained by squid during maceration, as well as the toxic elements that have a greater presence in squid (Cd and As). Therefore, maceration treatments may help to control the levels of polluting minerals in squid flesh. The consumption of macerated-cooked squid covered a relevant percentage of the DRI for Na, P, Zn, Mg and Mn. As maceration increases Na intake, this product should be consumed within a salt-balanced diet. The health risk associated with heavy metal intake would be almost negligible and mainly concerns Cd and As.

## Figures and Tables

**Table 1:** Fishing zones established by the Food and Agricultural Organization (FAO) and time course followed for obtaining whole squids.

	Fishing Zone	
	Mauritania	South Africa
FAO classification	No. 34	No. 47
FAO area coordinates	20.992732, −17.396224	−34.455970, 16.802051
Transportation in board to freezer	Frozen	Frozen
Capture and freezing date	13 December 2018	23 July 2018
Block’s reception date	12 February 2019	09 February 2019
Body weight average (±SD)	210 ± 28 g	243 ± 32 g

**Table 2:** Mineral content determined in the maceration medium used for squid processing.

Macrominerals	g/100 g	Microminerals	>1 mg/kg	Toxic Elements	mg/kg
Ca	<0.01	Al	3.60	As	0.48
K	0.02	Fe	3.25	Cd	<0.01
Mg	<0.01	Si	2.18	Pb	<0.01
Na	1.31	Sr	1.43	Hg	<0.01
P	<0.01	Zn	5.20		

Limits of Quantification (LoQ): 0.01 g/100 g (macrominerals), 0.1 mg/kg (Al, Fe and Si) and 0.01 mg/kg (rest of microminerals). Concentrations ranging 0.01–0.1 mg/kg (Be, Bi, Co, Cr, Cu, Li, Mo, Ni, Rb and Ti) are not shown.

**Table 3:** Proximate composition, basic volatile nitrogen and pH of raw, macerated and cooked squid flesh from two Atlantic fisheries.

		FAO 47				FAO 34				FAO 47 + 34					Overall		
		Raw		Cooked		Raw		Cooked		Raw		Cooked			Effects		
		M		M		M		M		M		M		*SEM*	Ma	C	Ma * C
Moisture	Untreated	81.10	^b^	77.07	^d^	78.56	^b^	76.59	^c^	79.83	^b^	76.83	^c^	*0.277*	***	***	***
g/100 g	Macerated	85.34	^a^	79.53	^c^	82.65	^a^	78.26	^b^	84.00	^a^	79.54	^b^				
Proteins	Untreated	15.86	^b^	18.63	^a^	18.81	^b^	19.93	^a^	17.34	^b^	19.28	^a^	*0.345*	***	***	^NS^
g/100 g	Macerated	11.06	^d^	13.98	^c^	14.19	^d^	16.34	^c^	12.63	^d^	15.16	^c^				
TBVN	Untreated	10.58	^c^	17.47	^a^	10.64	^d^	16.41	^b^	10.61	^c^	16.94	^a^	*0.332*	***	***	***
mg/100 g	Macerated	14.41	^bc^	16.33	^ab^	15.03	^c^	18.11	^a^	14.72	^b^	17.22	^a^				
Lipids	Untreated	2.01	^d^	3.35	^b^	1.77	^c^	2.67	^b^	1.89	^c^	3.01	^b^	*0.095*	***	***	*
g/100 g	Macerated	2.37	^c^	3.89	^a^	1.77	^c^	3.29	^a^	2.07	^c^	3.59	^a^				
Ash	Untreated	1.52	^c^	1.69	^b^	1.55	^c^	1.62	^c^	1.53	^c^	1.66	^bc^	*0.034*	***	***	***
g/100 g	Macerated	1.74	^b^	2.81	^a^	1.77	^b^	2.37	^a^	1.76	^b^	2.59	^a^				
pH	Untreated	6.75	^c^	7.15	^a^	6.56	^b^	7.13	^a^	6.66	^b^	7.14	^a^	*0.061*	***	**	***
	Macerated	6.96	^b^	7.11	^a^	7.21	^a^	7.10	^a^	7.09	^a^	7.10	^a^				

Abbreviations: FAO 47 and 34: Food and Agriculture Organization fishing zones No 47 and 34; M: mean; SEM: Standard Error of the Mean; Ma: Maceration; C: Cooking; TBVN: Total Basic Volatile Nitrogen. Means with different superscripts are different for *p* < 0.05 (samples from FAO 47, FAO 34 and FAO 47 + 34). Overall effects determined in whole sample (FAO 47 + 34): Levels of significance: *** *p* < 0.001; ** *p* < 0.01; * *p* < 0.05; ^NS^ *p* > 0.05.

**Table 4:** Mineral content of raw, macerated and cooked squid flesh from two Atlantic fisheries.

		FAO 47				FAO 34				FAO 47 + 34					Overall		
		Raw		Cooked		Raw		Cooked		Raw		Cooked			Effects		
Macrominerals (g/100 g)																	
		M		M		M		M		M		M		SEM	Ma	C	Ma*C
Ca	Untreated	0.03	^a^	0.01	^b^	0.02		0.02		0.03	^a^	0.01	^b^	*0.003*	^NS^	***	^NS^
	Macerated	0.03	^a^	0.02	^ab^	0.02		0.02		0.02	^ab^	0.02	^ab^				
K	Untreated	0.16	^a^	0.17	^a^	0.29	^a^	0.17	^b^	0.23	^a^	0.17	^b^	*0.011*	***	**	*
	Macerated	0.06	^b^	0.09	^b^	0.11	^c^	0.08	^c^	0.08	^c^	0.08	^c^				
Mg	Untreated	0.05	^a^	0.02	^c^	0.05	^a^	0.03	^b^	0.05	^a^	0.03	^c^	*0.002*	^NS^	***	***
	Macerated	0.04	^b^	0.03	^bc^	0.04	^a^	0.04	^a^	0.04	^b^	0.04	^b^				
Na	Untreated	0.48	^bc^	0.34	^c^	0.48	^b^	0.21	^c^	0.48	^b^	0.28	^c^	*0.037*	***	***	***
	Macerated	0.70	^a^	0.59	^ab^	0.80	^a^	0.36	^bc^	0.75	^a^	0.49	^b^				
P	Untreated	0.28	^a^	0.16	^b^	0.29	^a^	0.18	^b^	0.28	^a^	0.18	^b^	*0.015*	***	***	***
	Macerated	0.14	^b^	0.15	^b^	0.16	^b^	0.15	^b^	0.15	^b^	0.15	^b^				
S	Untreated	0.30	^a^	0.17	^b^	0.30	^a^	0.21	^b^	0.30	^a^	0.19	^b^	*0.011*	***	***	***
	Macerated	0.21	^b^	0.19	^b^	0.23	^b^	0.19	^b^	0.22	^b^	0.19	^b^				
Microminerals (>1 mg/kg)																	
Al	Untreated	3.72		2.14		0.74		0.97		2.23		1.89		*0.441*	^NS^	^NS^	^NS^
	Macerated	1.99		1.26		1.13		0.68		2.04		0.97					
Cu	Untreated	2.85	^a^	2.39	^a^	1.42	^ab^	1.79	^a^	2.03	^a^	2.09	^a^	*0.164*	***	*	^NS^
	Macerated	2.12	^a^	0.92	^b^	1.07	^b^	0.98	^b^	1.59	^a^	0.95	^b^				
Fe	Untreated	3.82	^a^	2.55	^ab^	1.46		2.34		2.64		2.44		*0.368*	^NS^	^NS^	^NS^
	Macerated	2.58	^ab^	1.36	^b^	1.35		2.04		1.96		1.70					
Mn	Untreated	2.57	^a^	1.26	^c^	2.63	^a^	2.48	^ab^	2.60	^a^	1.87	^b^	*0.145*	^NS^	***	*
	Macerated	2.05	^ab^	1.78	^bc^	2.21	^ab^	1.75	^b^	2.13	^ab^	1.77	^b^				
Si	Untreated	11.43		13.51		3.53	^b^	4.75	^ab^	7.48		9.13		*1.508*	^NS^	^NS^	^NS^
	Macerated	9.65		10.50		8.54	^a^	5.53	^ab^	9.09		8.01					
Sr	Untreated	3.44	^a^	2.35	^c^	2.54		3.02		2.30		2.81		*0.195*	^NS^	^NS^	^NS^
	Macerated	3.08	^ab^	2.64	^bc^	3.02		2.71		3.05		2.68					
Zn	Untreated	10.99		10.21		11.72	^a^	12.18	^a^	11.36	^a^	11.19	^a^	*0.562*	***	^NS^	^NS^
	Macerated	8.32		9.53		8.14	^b^	10.33	^ab^	8.23	^bc^	9.93	^ab^				
Toxic elements (mg/kg)																	
As	Untreated	1.53	^a^	1.45	^a^	1.37	^a^	1.11	^a^	1.45	^a^	1.28	^ab^	*0.104*	***	***	^NS^
	Macerated	1.14	^a^	0.51	^b^	0.90	^ab^	0.41	^b^	1.02	^b^	0.46	^c^				
Cd	Untreated	0.23	^b^	0.46	^a^	0.09	^bc^	0.17	^a^	0.16	^b^	0.32	^a^	*0.025*	***	***	^NS^
	Macerated	0.17	^b^	0.21	^b^	0.02	^d^	0.14	^ab^	0.10	^b^	0.17	^b^				
Hg	Untreated	0.02	^a^	0.01	^b^	0.02		0.01		0.02	^a^	0.01	^b^	*0.002*	*	**	**
	Macerated	0.01	^b^	0.01	^b^	0.01		0.01		0.01	^b^	0.01	^b^				
Pb	Untreated	0.02		0.05		<0.01	^b^	0.05	^a^	0.01	^b^	0.05	^ab^	*0.014*	^NS^	***	^NS^
	Macerated	0.01		0.08		<0.01	^b^	0.07	^a^	0.01	^b^	0.07	^a^				

Abbreviations: FAO 47 and 34: Food and Agriculture Organization fishing zones No 47 and 34; M: mean; SEM: Standard Error of the Mean; Ma: Maceration; C: Cooking. Means with different superscripts are different for *p* < 0.05 (samples from FAO 47, FAO 34 and FAO 47 + 34). Overall effects determined in whole sample (FAO 47 + 34): Levels of significance: *** *p* < 0.001; ** *p* < 0.01; * *p* < 0.05; ^NS^ *p* > 0.05. Limits of Quantification (LoQ): 0.01 g/100 g (macrominerals), 0.1 mg/kg (Al, Fe and Si) and 0.01 mg/kg (rest of microminerals). Mineral concentrations ranging 0.01–0.1 mg/kg (B, Co, Cr, Cu, Li, Ni, Rb, Se, Ti and Tl) are not shown.

**Table 5:** Total intake and percent covered (in parentheses) of the Daily Recommended Intake (DRI) for selected minerals through consumption of 100 g cooked squid by an adult consumer weighing 60 kg.

		FAO 47		FAO 34		FAO 47 + 34		
		M		M		M		DRIs
Macrominerals (mg)								
Ca	Untreated	27	(2.7)	16	(1.6)	22	(2.2)	1000 mg
	Macerated	27	(2.7)	11	(1.1)	19	(1.9)	
K	Untreated	163	(3.5)	91	(1.9)	127	(2.7)	4700 mg
	Macerated	35	(0.7)	170	(3.6)	102	(2.2)	
Mg	Untreated	53	(15.1)	32	(9.1)	42	(12.1)	350 mg
	Macerated	38	(10.9)	23	(6.6)	30	(8.7)	
Na	Untreated	475	(20.7)	293	(12.7)	384	(16.7)	2300 mg
	Macerated	704	(30.6)	594	(25.8)	649	(28.2)	
P	Untreated	121	(17.3)	176	(25.1)	149	(21.2)	700 mg
	Macerated	150	(21.4)	150	(21.4)	150	(21.4)	
S	Untreated	169		212		191		(1)
	Macerated	195		187		191		
Microminerals (μg)								
Cu	Untreated	92	(0.9)	98	(1.0)	95	(1.0)	10,000 μg
	Macerated	239	(2.4)	179	(1.8)	209	(2.1)	
Fe	Untreated	255	(0.6)	204	(0.5)	230	(0.5)	45,000 μg
	Macerated	136	(0.3)	234	(0.5)	185	(0.4)	
Mn	Untreated	178	(8.7)	175	(8.5)	177	(8.6)	2050 μg
	Macerated	126	(6.1)	248	(12.1)	187	(9.1)	
Zn	Untreated	953	(9.5)	1033	(10.3)	993	(9.9)	10,000 μg
	Macerated	1020	(10.2)	1218	(12.2)	1119	(11.2)	

Abbreviations: FAO 47 and FAO 34: Food and Agriculture Organization fishing zones N° 47 and 34. DRIs published by the Institute of Medicine (United States of America) [34]. (1) Not established for S.

**Table 6:** Estimated Weekly Intake (EWI) and equivalent per cent coverage of the Provisional Tolerable Weekly Intake (% PTWI) for As, Pb, Cd and Hg through consumption of cooked squid.

			FAO 47		FAO 34		FAO 47 + 34	
	PTWI (μg Per kg Body wt and wk)		EWI	% PTWI	EWI	% PTWI	EWI	% PTWI
As	15 μg	Untreated	1.66	11.1	1.27	8.5	1.38	9.8
		Macerated	0.68	3.9	0.47	3.1	0.50	3.5
Cd	2.5 μg	Untreated	0.52	20.8	0.20	8.0	0.36	14.4
		Macerated	0.23	9.2	0.16	6.4	0.20	7.8
Hg	7 μg	Untreated	0.01	0.1	0.01	0.1	0.01	0.1
		Macerated	0.01	0.1	0.01	0.1	0.01	0.1
Pb	25 μg	Untreated	0.06	0.2	0.06	0.2	0.06	0.2
		Macerated	0.09	0.4	0.08	0.1	0.09	0.2

Abbreviations: FAO 47 and FAO 34: Food and Agriculture Organization fishing zones N° 47 and 34. wt; weight; wk: week; b.w.; body weight; w;w; wet weight. EWI = (C × FIR × 7) / WAB. EWI: Estimated Weekly Intake (μg/kg b.w.); C: metal concentration (μg/g w.w.); FIR: Food Ingestion Rate for cephalopods (9.8 g per person and day); 7: weekly basis; and WAB: average consumer body weight (60 kg).

## Data Availability

Not applicable.
